# Plasma acylcarnitine profiling reveals heart failure-specific fatty acid oxidation signature in chronic kidney disease

**DOI:** 10.1016/j.jlr.2026.101072

**Published:** 2026-05-29

**Authors:** Farsad Afshinnia, Thekkelnaycke M. Rajendiran, Jaeman Byun, Subramaniam Pennathur

**Affiliations:** 1 Department of Internal Medicine-Nephrology, University of Michigan, Ann Arbor, MI; 2 Department of Pathology, University of Michigan, Ann Arbor, MI, USA; 3 Departments of Internal Medicine-Nephrology and Molecular and Integrative Physiology, University of Michigan, Ann Arbor, MI

**Keywords:** Acylcarnitines, heart failure, chronic kidney disease, mass spectrometry

## Abstract

Acylcarnitines are key intermediates in fatty acid oxidation (FAO). Chronic kidney disease (CKD) and heart failure (HF) both alter FAO. However, it is unclear whether specific FAO changes are associated with CKD coupled with HF. Aim is to investigate alterations in various chain acylcarnitines in patients with CKD with and without HF and examine their independent associations. In a case-control study at the University of Michigan (2010–2022), 562 participants with HF and available plasma samples were selected and compared with 461 participants without HF, frequency matched by CKD stage. Plasma samples were retrieved for mass spectrometry-based acylcarnitine quantification. Mean age (± standard deviation) was 65 ± 14 years in HF and 54 ± 14 years in those without HF. We observed a significant increase in short-chain acylcarnitine (SCAC) and medium-chain acylcarnitine (MCAC) but a decrease in long-chain acylcarnitines (LCACs) due to the worsening stage of CKD in the absence of HF. In patients with HF, the slope of changes in SCAC and LCAC was mitigated, whereas the increase in MCAC due to worsening CKD stage was greater than that in the absence of HF. The mean SCAC, MCAC, and LCAC scores were significantly different at CKD stage 4 in HF versus those without HF (*P* ≤ 0.002). These findings suggest that CKD with HF is characterized by impaired β-oxidation of MCAC and LCAC and greater myocardial utilization of short-chain fatty acids. Inefficient β-oxidation coupled with the accumulation of MCAC and LCAC may in part explain the poorer outcomes in the HF–CKD complex.

Over 37 million people have chronic kidney disease (CKD) in the United States https://www.cdc.gov/kidneydisease/pdf/2019_National-Chronic-Kidney-Disease-Fact-Sheet.pdf. In CKD, cardiovascular disorders are the leading causes of mortality and morbidity, with heart failure (HF) being the leading cardiovascular complication (1). Approximately 17%–50% of the patients with CKD develop HF, and approximately 40%–50% of the patients with HF develop CKD (2). Patients with CKD and HF experience exponentially increased mortality and hospitalization rate (3, 4, 5). Hence, the coexistence of the two conditions reflects a high-risk large segment of the patient population becoming a high priority in healthcare research. Traditional risk factors for HF do not fully explain the risk of HF in CKD patients (6). In contrast, impairment of fatty acid oxidation (FAO) takes place in both CKD (7) and HF (8). It is yet unclear how FAO is affected by more advanced stages of CKD in patients with HF than in those without HF. Demonstration of HF-specific alterations in FAO may enhance the insight into novel pathophysiology promoting the clinical syndrome of HF in CKD.

Acylcarnitines (AC) are esterified carnitines that play a crucial role in transporting fatty acids into the mitochondria for ATP generation via β-oxidation. They are classified to short-chain acylcarnitine (SCAC), medium-chain acylcarnitine (MCAC), and long-chain acylcarnitine (LCAC) based on their class specific similarities in fatty acid metabolism, mitochondrial transport mechanisms, and their relevance to metabolic disorders (9). SCAC are byproducts of both long-chain fatty acids, ketoacids (C2 and C4), and branched-chain amino acid metabolism (C3 and C5). Additionally, they may be derived from dietary and microbial sources (C2-C5). MCAC and LCAC are mainly driven by endogenous metabolic pathways of β-oxidation of long-chain fatty acids. LCAC are gateways of free fatty acids to mitochondria, and their low abundance reflects reduced energy production via β-oxidation. In HF, altered circulating AC levels are linked to poor cardiovascular outcomes (10, 11, 12, 13, 14). CKD progression also decreases LCAC (15). However, it is still unclear how the coexistence of the CKD–HF complex impacts various lengths of AC and hence the efficiency of downstream β-oxidation. We aimed A) to compare the alterations in the abundance of various chain AC across predialysis CKD stages in patients with and without HF and B) to examine the independent association of various chain AC with CKD stages in patients with and without HF. We hypothesized that A) HF-specific alterations in SCAC, MCAC, and LCAC by CKD progression differ from those in their counterparts without HF and that B) the alterations are independent of traditional risk factors, therapeutic management, or clinical characteristics.

## Materials and Methods

### Participants

This study was a case-control observation of participants with and without HF with available stored plasma samples nested in patients under the care of the University of Michigan (UM) from January 2010 to April 2022. The study was approved by the Institutional Review Board (HUM00196702) and adhered to the Declaration of Helsinki. Patients receiving care at the UM provided opportunities to donate biosamples for future research after obtaining informed consent. The samples were stored at the Central Biorepository at UM. To build the cohort, we searched the data warehouse to identify eligible participants with HF. Cases (n = 562) were defined as participants with HF screened by International Classification of Disease (ICD) codes and verified by established care for HF management under the care of a cardiologist with the availability of at least 200 μl of plasma samples at the UM Central Biorepository. The control group (n = 461) was defined as participants without HF with 200 μl of plasma samples, frequency-matched with the case group by CKD stage. CKD stage was the matching variable. The inclusion criteria were age ≥20 years and estimated glomerular filtration rate (eGFR) ≥15 ml/min at the time of sample collection, without any sex or race restriction. The exclusion criteria were HF due to valvular disease, septal defect, infiltrative diseases (sarcoidosis, amyloidosis), chemotherapy, and viral and peripartum cardiomyopathy, aimed at enriching the participants with HF with metabolic etiology as opposed to other known etiologies of HF. CKD classification was according to Kidney Disease: Improving Global Outcomes (KDIGO) definition using eGFR cutoffs for stage classification study (stage 1: ≥90; stage 2: 60–89; stage 3: 30–59; stage 4: 15–29 ml/min/1.73 m^2^) using the Chronic Kidney Disease Epidemiology Collaboration (CKD-EPI) formula for eGFR estimation (16).

### HF diagnosis

For screening, we used the ICD-9 and ICD-10 codes to search the data warehouse to identify eligible participants with HF (supplemental Table S1). To validate the diagnosis, concordance on diagnosis by two physicians were required, the referring physician and the cardiologist who established continued care for HF management. Plasma samples were retrieved when patients were clinically stable with a compensated heart function. Using two-dimensional echocardiogram, cases of heart failure were categorized to heart failure with preserved ejection fraction (HFpEF, EF ≥ 50%) and heart failure with reduced ejection fraction (HFrEF, EF<50%). Subsequently, it was further classified by left ventricular (LV) geometry into normal LV geometry, concentric LV remodeling (cLVR), concentric LV hypertrophy (cLVH), and eccentric LV hypertrophy (eLVH). LV mass (LVM) was calculated from measurements of other LV dimensions using the formula: LVM = [1.04 × (IVST + PWT + LVEDD)3 – LVEDD3], where IVST, posterior wall thickness (PWT), and LVEDD stand for interventricular septal thickness, posterior wall thickness, and LV end-diastolic dimension, respectively (17). LV mass index (LVMI) was defined as LV mass standardized by BSA. LV hypertrophy (LVH) was defined as LVMI greater than 110 g/m^2^ in females and greater than 130 g/m^2^ in males (18, 19, 20). Relative wall thickness (RWT) was calculated as (2 × PWT)/LVEDD. cLVR was defined as normal LVMI with an RWT of 0.44 or greater. cLVH was defined as LVH with an RWT of 0.44 or greater, and eLVH as LVH with an RWT of less than 0.44 (21, 22). In our unit, the intraclass correlation coefficient was 0.98 for LVEDD, 0.94 for LVESD, 0.94 for IVST, 0.97 for PWT, 0.98 for LVMI, and 1.0 for EF <50%, and coefficients of variation was 1.8% for LVEDD, 5.8% for LVESD, 4.8% for IVST, 5.2% for PWT, and 7.2% for LVMI (22).

### Data collection

Baseline characteristics, including demographics, anthropometrics, blood pressure, comorbid conditions, medications, echocardiograms, and laboratory findings at the time of sample collection (±3 months), were retrieved from the UM data warehouse. Research protocol-based uniform sample collection applied to all participants standardized sample collection process and minimized allocation bias. The identified samples stored at −80 °C in the biorepositories were retrieved and transported to −80 °C at the research laboratory until the time of AC extraction. Five hundred microliters of methanol:chloroform:water (8:1:1) and 1 μl of AC mixed internal standards were added to 25 μl of thawed plasma samples for AC extraction. The authenticated internal standards were purchased from Cambridge Isotope Laboratories Inc (supplemental Table S2). The supernatant was dried under nitrogen and resuspended in 50 μl water:acetonitrile (95:5) mixed with 15 mM NH4OAc. Five microliters was injected into the Agilent 6490 Triple quadrupole tandem mass spectrometer (Agilent) for data acquisition in multiple reaction monitoring mode, as reported previously (15, 18, 19). For quality assurance and quality control and to minimize bias from machine drift, we randomized the samples to a new worklist and ran a reference pool of the samples at the beginning and after each of the 10 samples to assess reproducibility and drift in mass spectrometry performance over time. We quantified L-carnitine and 28 AC (supplemental Table S3).

### Quality control and quality assurance

The quality assurance/quality control of mass spectrometry required randomization of samples to a randomized new sequence prior to mass spectrometry data acquisition, sequential run of 10 reference pools at the start of the run to ensure reproducibility of instrument, and run of pooled samples derived from the entire study cohort were injected at the start and after every 15 samples to monitor instrument drift. The coefficients of variation for the internal standards were calculated to assess measurement stability. The values supported assay reproducibility and ruled out the need for additional batch-effect normalization. The coefficients of variation for AC measurements are reported in supplemental Table S2.

### Statistical analysis

The step by step computational pipeline included the following: (i) raw mass spectrometry signals for each AC were normalized to the corresponding authenticated internal standard for absolute quantification; (ii) values were sum-normalized within each sample to express each AC as a fraction of total AC, which captures relative class-level abundance and avoids the misleading interpretations that arise when absolute concentrations alone are compared between samples with differing total AC pools; (iii) sum-normalized proportions (constrained between 0 and 1) were logit-transformed into an unbounded continuous scale (–∞ to +∞) to enable linear modeling and stabilize variance; and (iv) values were z-score standardized to allow side-by-side comparison in heatmaps, since absolute AC concentrations span μmol/L to nmol/L.

For description, we used the mean and standard deviation (SD) for normally distributed continuous variables, median and interquartile range for skewed continuous variables, and counts with frequencies for categorical variables. Linear regression models were used to assess the trend of variables across CKD stages. For class-level comparisons, we used mixed general linear models in long-format data, with the corresponding ≤ C5, C6–C14, or C16–C20 AC values as the dependent variable and the clinical and demographic characteristics as fixed covariates. To assess the independence of AC alterations across CKD stages from the covariates, we ran unadjusted to fully adjusted generalized linear mixed multinomial logistic regression analyses with CKD stages as the dependent variable and HF status and AC class as predictors. The rationale for modeling was to test if the AC alterations by CKD stage were independent of imbalanced baseline covariates and their differences at various CKD stages. Therefore, the covariates that had significant changes in their descriptive statistics from stage 1 to stage 4 were selected to be adjusted for. We applied different models from unadjusted to fully adjusted models with increasing level of adjustment to allow assessing effect of confounders, demonstrate model stability, and understand how specific set of variables contribute to observed relationships. Model 1 was unadjusted model. Model 2 was model 1 + age, + sex, + Race. Model 3 was model 2 + alcohol use + Charlson score + hemoglobin + albumin + diabetes. Model 4 was model 3 + BMI + smoking + insulin use + noninsulin hypoglycemic agents + statin use + steroid use + antihypertensive use + diuretic use. The coefficients of variation for the AC measurements are presented in supplemental Table S2.

### Sample size and study power

All participants with HF from January 2010 to April 2022 who had at least 200 uL of stored plasma samples who met the inclusion and exclusion criteria were selected and matched by participants without HF and available plasma samples matched by CKD stage. Accordingly, 562 participants in case group and 461 participants in control group provide over 95% power to detect 0.1 unit standardized mean difference by study groups.

## Results

### Baseline characteristics

The baseline characteristics of the participants with and without HF according to eGFR categories are shown in Table 1. In all participants, there was a graded increase in mean age, Charlson comorbidity index (20), and median urine protein-creatinine ratio but a graded decrease in mean hemoglobin, serum albumin, total cholesterol, and LDL by worsening eGFR categories (Table 1, *P* < 0.001), irrespective of HF. There was a significant increase in the percentage of diabetes, use of statins, and insulin but a decrease in alcohol use by worsening eGFR categories. In participants without HF, there was also a significant increase in total serum triglycerides, use of antihypertensive agents, steroids, and noninsulin hypoglycemic agents by worsening eGFR categories. There was no significant trend in sex, race, smoking, mean BMI, HDL, or use of fibrates according to eGFR categories. Compared to those without HF, patients with HF had significantly different baseline age, hemoglobin, antihypertensive agents, and diuretics (at all stages); use of steroids and noninsulin hypoglycemic agents (at stage 1); sex, white race, smoking, LDL, and HDL (at stage 2); triglycerides (at stage 3); Charlson score and BMI (at stages 1 and 2); statin use (at stages 1–3); diabetes, albumin, and total cholesterol (at stages 2–4).

**Table 1:** Baseline characteristics of participants with and without heart failure by categories of eGFR

Variables	eGFR ≥90 ml/min		eGFR 60–89 ml/min		eGFR 30–59 ml/min		eGFR 15–29 ml/min	
	-HF (n = 81)	+HF (n = 100)	-HF (n = 199)	+HF (n = 204)	-HF (n = 146)	+HF (n = 206)	-HF (n = 35)	+HF (n = 52)
Age (years)^a^!	42 ± 13	52 ± 14^c^	53 ± 13	63 ± 12^c^	61 ± 12	70 ± 12^c^	57 ± 13	74 ± 12^c^
Male sex (%)	33 (40.7)	59 (59.0)	71 (35.7)	130 (61.7)^c^	71 (48.6)	121 (58.7)	18 (51.4)	23 (44.2)
White race (%)	69 (85.2)	82 (82.0)	188 (94.5)	166 (81.4)^c^	130 (89.0)	178 (86.4)	34 (97.1)	44 (84.6)
Smoking								
Current (%)	3 (3.7)	11 (11.0)	6 (3.0)	14 (16.9)^c^	7 (4.8)	8 (3.9)	1 (2.9)	2 (3.8)
Former (%)	29 (35.8)	40 (40.0)	82 (41.2)	101 (49.5)	61 (41.8)	115 (55.8)	13 (37.1)	21 (40.4)
Never (%)	49 (60.5)	49 (49.0)	111 (55.8)	85 (41.7)	78 (53.4)	80 (38.8)	21 (60.0)	29 (55.8)
Alcohol intake (%)^a^!	39 (48.1)	47 (47.0)	90 (45.2)	88 (43.1)	55 (37.7)	73 (35.4)	9 (25.7)	12 (23.1)
Diabetes (%)^a^!	38 (46.9)	28 (28.0)	106 (53.3)	81 (39.7)^b^	139 (95.2)	117 (56.8)^c^	33 (94.3)	30 (57.7)^c^
Charlson score [IQR]^a^!	1 [0.5–3]	2 [0–3]^c^	3 [1–5]	6 [3–9]^c^	6 [5–11]	8 [5–14]	7 [5–13]	10 [6–16]
Body mass index (kg/m^2^)	34.6 ± 6.6	31.9 ± 8.8^b^	34.2 ± 6.3	32.4 ± 8.1^b^	34.1 ± 6.7	33.1 ± 8.4	31.5 ± 6.9	30.2 ± 6.1
Hemoglobin (g)^a^!	13.7 ± 1.3	13.0 ± 1.6^c^	13.5 ± 1.7	12.7 ± 2.1^c^	13.2 ± 1.9	11.8 ± 2.0^c^	12.2 ± 1.9	10.7 ± 1.9^c^
Albumin (g/dl)^a^!	4.4 ± 0.4	4.1 ± 0.5	4.2 ± 0.4	4.0 ± 0.6^c^	4.2 ± 0.5	3.9 ± 0.5^c^	4.0 ± 0.5	3.6 ± 0.4^c^
Total cholesterol (mg/dl)^a^!	181 ± 43	171 ± 42	180 ± 38	161 ± 41^c^	180 ± 45	160 ± 44^c^	169 ± 45	146 ± 37^b^
LDL (mg/dl)^a^!	98 ± 32	92 ± 34	95 ± 34	87 ± 32^b^	88 ± 34	84 ± 34	74 ± 27	73 ± 28
HDL (mg/dl)	50 ± 14	51 ± 19	53 ± 16	47 ± 16^c^	48 ± 14	47 ± 17	48 ± 18	48 ± 17
Triglycerides (mg/dl)^a^	132 ± 104	148 ± 116	143 ± 99	153 ± 108	205 ± 156	158 ± 114^c^	170 ± 143	147 ± 101
Medications:								
Antihypertensive (%)^a^	12 (14.8)	88 (88.0)^c^	76 (38.2)	180 (88.2)^c^	93 (63.7)	191 (92.7)^c^	24 (68.6)	51 (98.1)^c^
Diuretics (%)^a^	3 (3.7)	82 (82.0)^c^	36 (18.1)	172 (84.3)^c^	56 (38.4)	183 (88.8)^c^	15 (42.9)	47 (90.4)^c^
Statins (%)^a^!	10 (12.3)	40 (40.0)^c^	49 (24.6)	134 (65.7)^c^	68 (46.6)	144 (69.9)^c^	19 (54.3)	40 (76.9)
Fibrates (%)	1 (1.2)	2 (2.0)	8 (4.0)	21 (10.3)	9 (6.2)	24 (11.7)	0 (0)	4 (7.7)
Steroids (%)^a^	9 (11.1)	33 (33.0)^c^	35 (17.6)	55 (27.0)	42 (28.8)	72 (35.0)	16 (45.7)	23 (44.2)
Insulin (%)^a^!	11 (13.6)	23 (23.0)	56 (28.1)	65 (31.9)	86 (58.9)	106 (51.5)	21 (60.0)	27 (51.9)
Noninsulin HA (%)^a^	2 (2.5)	16 (16.0)^b^	23 (11.6)	39 (19.1)	27 (18.5)	46 (22.3)	5 (14.3)	10 (19.2)
UPCR [IQR]^a^!	0.09 [0.05–0.19]	0.08 [0.04–0.21]	0.10 [0.06–0.22]	0.10 [0.05–0.24]	0.19 [ 0.08–0.67]	0.12 [0.07–0.47]	0.37 [0.16–1.55]	0.39 [0.08–1.13]

Values are Mean ± standard deviation or count (percentage), except for the Charlson score and UPCR, which are median and interquartile range (IQR).

ACEI, angiotensin-converting enzyme inhibitor; ARB, angiotensin receptor blocker; HDL, high-density lipoprotein; HF, heart failure; LDL, low-density lipoprotein; MRA, mineralocorticoid receptor antagonist; UPCR, urine protein creatinine ratio.

a *P* Trend by CKD stage with no heart failure ≤0.002. *P* Trend by CKD stage in heart failure ≤0.002; In patients without heart failure, UPCR was available only for 14 participants with eGFR ≥90 ml/min, 40 with eGFR 60–89 ml/min, 89 with eGFR 30–59 ml/min, and 32 with eGFR 15–29 ml/min. In patients with heart failure, UPCR was available only for 26 patients with eGFR ≥90 ml/min, 40 with eGFR 60–89 ml/min, 94 with eGFR 30–59 ml/min, and 35 with eGFR 15–29 ml/min.

b *P* ≤ 0.01 comparing HF with no HF within CKD stage.

c *P* ≤ 0.001 comparing HF with no HF within CKD stage. Values are mean ± standard deviation, or count and percentage. For UPCR and Charlson score, values are median and interquartile range.

### Heart failure subtypes

There were 303 patients (53.9%) with HFpEF and 259 (46.1%) with HFrEF. Distribution of LV dimensions and configurations by CKD stage are shown in Table 2. In HFpEF, there was a significant increase in intraventricular septal thickness, PWT, and percentage of cLVR and CLVH by worsening stage of CKD (*P* < 0.01), whereas in HFrEF, there was a significant increase in LVMI by worsening stage of CKD. Other cardiac dimensions and configurations did not reach statistical significance.

**Table 2:** Distribution of cardiac dimensions and configurations by heart failure subtype and stages of chronic kidney disease in patients with heart failure

HFpEF	CKD 1	CKD 2	CKD 3	CKD 4
	(n = 53)	(n = 97)	(n = 119)	(n = 34)
IVS (mm)^a^	10.0 ± 2.3	10.7 ± 3.0	11.0 ± 2.4	11.3 ± 2.0
PWT (mm)^a^	9.8 ± 1.9	10.3 ± 2.8	10.6 ± 2.2	11.2 ± 2.3
LVEDD (mm)	49 ± 7	49 ± 7	48 ± 7	47 ± 7
LVESD (mm)	32 ± 7	33 ± 7	32 ± 7	31 ± 7
LVM (gm)	226 ± 76	245 ± 90	244 ± 79	252 ± 78
LVMI (g/m^2^)	112 ± 33	119 ± 43	119 ± 34	131 ± 36
EF (%)	61 ± 6	62 ± 6	62 ± 7	62 ± 7
nLV (%)	24 (45.3)	43 (44.8)	41 (34.7)	7 (20.6)
cLVR, cLVH (%)^a^	19 (35.8)	36 (37.5)	58 (49.2)	21 (61.8)
eLVH (%)	10 (18.9)	17 (17.7)	19 (16.1)	6 (17.6)
HFrEF	(n = 47)	(n = 107)	(n = 87)	(n = 18)
IVS (mm)	9.5 ± 2.0	10.1 ± 1.9	10.1 ± 1.4	11.1 ± 2.0
PWT (mm)	9.8 ± 1.9	9.7 ± 2.3	9.6 ± 2.2	11.0 ± 2.2
LVEDD (mm)	60 ± 10	62 ± 11	63 ± 12	62 ± 10
LVESD (mm)	48 ± 14	51 ± 12	54 ± 13	50 ± 13
LVM (gm)	311 ± 127	339 ± 126	350 ± 133	376 ± 114
LVMI (g/m^2^)^a^	147 ± 56	162 ± 55	167 ± 60	188 ± 54
EF (%)	29 ± 11	31 ± 10	27 ± 10	27 ± 9
nLV (%)	16 (34.0)	26 (24.3)	25 (28.7)	1 (5.6)
cLVR, cLVH (%)	5 (10.6)	15 (14.0)	6 (6.9)	3 (16.7)
eLVH (%)	26 (55.3)	66 (61.7)	56 (64.4)	14 (77.8)

CKD, chronic kidney disease; cLVR, concentric left ventricular remodeling; EF, ejection fraction; eLVH, eccentric left ventricular hypertrophy; HFpEF, heart failure with preserved ejection fraction; HFrEF, heart failure with reduced ejection; IVS, interventricular septal thickness; LVEDD, left ventricular end-diastolic dimension; LVESD, left ventricular end-systolic dimension; LVM, left ventricular mass; LVMI, left ventricular mass index; nLV, normal left ventricular configuration; PWT, posterior wall thickness.

Values are mean ± SD, or count and percentage.

a *P* ≤ 0.01 linear trend by CKD stage.

### AC levels

**Fig. 1:**
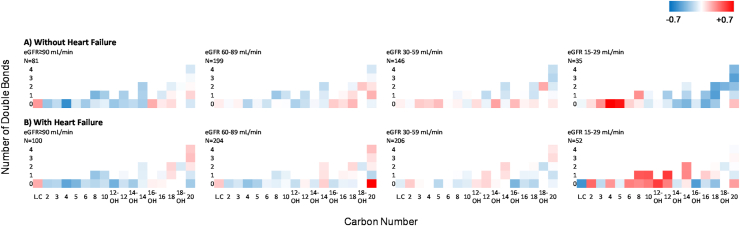
Differential mean lipid heat map illustrating the standardized mean level of each acylcarnitine at various eGFR categories by heart failure in participants with and without diabetes. Panel A refers to participants without heart failure, whereas Panel B refers to participants with heart failure.

**Fig. 2:**
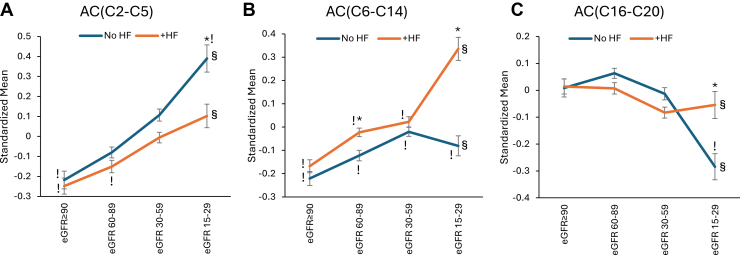
Comparing mean level of short chain (C2-C5), medium chain (C6-C14), and long chain (C16-C20) acylcarnitines by heart failure and eGFR categories. Panel A refers to alterations of short-chain (C2-C5), Panel B to medium-chain (C6-C14), and Panel C to long-chain (C16-20) acylcarnitines. HF: Heart failure; ∗*P* ≤ 0.002 compared with no HF within the same eGFR category; !*P* ≤ 0.002 interaction term for the corresponding group compared with heart failure at CKD stage 4 (eGFR 15–29 ml/min) as reference group using 2-sided Dunnett test adjusted for multiplicity; §*P* ≤ 0.001 trend by eGFR categories. Values are mean and standard errors.

Mean of individual AC by CKD stage and HF is shown in supplemental Table S4 (concentration), supplemental Table S5 (standardized) and Fig. 1 (Standardized). The mean of SCAC significantly increased with worsening eGFR in patients with and without HF (Fig. 2A). The slope of increase of SCAC by CKD stage in participants without HF was significantly higher as compared to those with HF, which resulted in a significantly higher SCAC at eGFR<30 ml/min in HF compared with no HF (*P* < 0.001). The slope of increased MCAC by CKD was significantly higher in HF compared to no HF (Fig. 2B), so that the mean MCAC was significantly higher at eGFR 60–89 ml/min and more so at eGFR<30 ml/min in those with HF than in those without (*P* < 0.001). LCAC significantly declined in both groups with and without HF, but the slope of the decline was mitigated in the HF group, so that the mean LCAC was significantly lower at eGFR<30 ml/min in patients without HF than in those with HF (Fig. 2C, *P* < 0.001).

### Association of AC levels with various eGFR categories by HF

To investigate the odds of AC at various CKD stages in participants with and without HF, we applied unadjusted to fully adjusted multinomial logistic regression.

#### Short-chain acylcarnitine

In participants without HF, worsening stages of CKD (eGFR <90 ml/min) were associated with higher odds of increasing SCAC than normal kidney function (eGFR ≥90 ml/min). In the fully adjusted model, CKD stage 3 (eGFR 30–59 ml/min) was associated with 1.24-fold (95% CI 1.08–1.41, *P* = 0.002) and stage 4 with 1.81-fold (95% CI: 1.48–2.21, *P* < 0.001)higher odds of increasing the abundance of SCAC as compared to eGFR ≥90 ml/min (Table 3). Similarly, in participants with HF, CKD stages were associated with a higher abundance of SCAC than normal kidney function. In the fully adjusted model, CKD stage 2 was associated with 1.21-fold (95 CI: 1.07–1.35, *P* = 0.002), stage 3 with 1.33-fold (95% CI: 1.17–1.51, *P* < 0.001); and stage 4 with 1.46-fold (95% CI: 1.25–1.70, *P* < 0.001) higher odds of increasing the abundance of SCAC as compared to eGFR ≥90 ml/min (Table 3).

**Table 3:** Association of acylcarnitine levels (each 1 standard deviation change) by heart failure at various categories of eGFR compared with eGFR ≥90 ml/min

	Model 1		Model 2		Model 3		Model 4	
	OR (95% CI)	*P*	OR (95% CI)	*P*	OR (95% CI)	*P*	OR (95% CI)	*P*
AC C2–C5								
No HF								
eGFR≥90 ml/min	1 (Reference)		1 (Reference)		1 (Reference)		1 (Reference)	
eGFR 60–89 ml/min	1.16 (1.04–1.30)	0.010	1.08 (0.92–1.21)	0.148	1.06 (0.95–1.19)	0.269	1.07 (0.95–1.19)	0.265
eGFR 30–59 ml/min	1.44 (1.28–1.62)	<0.001	1.33 (1.17–1.51)	<0.001	1.24 (1.08–1.41)	0.002	1.29 (1.13–1.47)	<0.001
eGFR 15–29 ml/min	1.89 (1.61–2.32)	<0.001	1.86 (1.56–2.22)	<0.001	1.83 (1.50–2.23)	<0.001	1.89 (1.54–2.30)	<0.001
With HF								
eGFR≥90 ml/min	1 (Reference)		1 (Reference)		1 (Reference)		1 (Reference)	
eGFR 60–89 ml/min	1.11 (1.01–1.22)	0.025	1.22 (1.10–1.36)	<0.001	1.21 (1.08–1.35)	0.001	1.19 (1.06–1.33)	0.004
eGFR 30–59 ml/min	1.27 (1.15–1.40)	<0.001	1.43 (1.27–1.60)	<0.001	1.33 (1.17–1.50)	<0.001	1.32 (1.17–1.49)	<0.001
eGFR 15–29 ml/min	1.43 (1.24–1.64)	<0.001	1.59 (1.37–1.85)	<0.001	1.44 (1.23–1.67)	<0.001	1.48 (1.27–1.73)	<0.001
AC C6–C14								
No HF								
eGFR≥90 ml/min	1 (Reference)		1 (Reference)		1 (Reference)		1 (Reference)	
eGFR 60–89 ml/min	1.11 (1.02–1.20)	0.013	1.05 (0.97–1.13)	0.275	1.07 (0.99–1.16)	0.088	1.09 (1.01–1.19)	0.030
eGFR 30–59 ml/min	1.28 (1.17–1.39)	<0.001	1.18 (1.07–1.29)	<0.001	1.18 (1.07–1.30)	<0.001	1.19 (108–1.31)	<0.001
eGFR 15–29 ml/min	1.19 (1.06–1.35)	0.005	1.07 (0.94–1.23)	0.300	1.00 (0.87–1.16)	0.967	1.09 (0.94–1.26)	0.262
With HF								
eGFR≥90 ml/min	1 (Reference)		1 (Reference)		1 (Reference)		1 (Reference)	
eGFR 60–89 ml/min	1.15 (1.07–1.23)	<0.001	1.12 (1.04–1.21)	0.002	1.09 (1.01–1.17)	0.028	1.07 (0.99–1.16)	0.073
eGFR 30–59 ml/min	1.20 (1.19–1.28)	<0.001	1.10 (1.02–1.19)	0.019	1.03 (0.95–1.12)	0.469	1.00 (0.92–1.09)	0.936
eGFR 15–29 ml/min	1.61 (1.47–1.77)	<0.001	1.42 (1.29–1.58)	<0.001	1.29 (1.16–1.43)	<0.001	1.27 (1.14–1.41)	<0.001
AC C16–C20								
No HF								
eGFR≥90 ml/min	1 (Reference)		1 (Reference)		1 (Reference)		1 (Reference)	
eGFR 60–89 ml/min	1.07 (0.99–1.16)	0.101	1.09 (1.02–1.18)	0.019	1.15 (1.06–1.24)	<0.001	1.17 (1.08–1.26)	<0.001
eGFR 30–59 ml/min	0.98 (0.90–1.06)	0.587	1.00 (0.92–1.09)	0.959	1.11 (1.02–1.22)	0.022	1.08 (0.99–1.18)	0.102
eGFR 15–29 ml/min	0.75 (0.67–0.84)	<0.001	0.72 (0.64–0.82)	<0.001	0.74 (0.65–0.86)	<0.001	0.76 (0.66–0.87)	<0.001
With HF								
eGFR≥90 ml/min	1 (Reference)		1 (Reference)		1 (Reference)		1 (Reference)	
eGFR 60–89 ml/min	0.99 (0.93–1.06)	0.834	0.95 (0.88–1.03)	0.197	0.94 (0.86–1.01)	0.099	0.94 (0.86–1.01)	0.107
eGFR 30–59 ml/min	0.91 (0.85–0.97)	0.006	0.85 (0.78–0.92)	<0.001	0.85 (0.78–0.92)	<0.001	0.85 (0.78–0.92)	<0.001
eGFR 15–29 ml/min	0.93 (0.88–1.03)	0.161	0.86 (0.77–0.95)	0.003	0.89 (0.80–0.99)	0.035	0.89 (0.80–0.99)	0.029

AC, acylcarnitine; HF, heart failure; OR, odds ratio; 95% CI, 95% confidence interval; model 1, unadjusted; model 2, model 1 + age, + sex, + race; model 3 = model 2 + alcohol use + Charlson score + hemoglobin + albumin + diabetes; model 4 = model 3 + BMI + smoking + insulin use + noninsulin hypoglycemic agents + statin use + steroid use + antihypertensive use + diuretic use (fully adjusted).

#### Medium-chain acylcarnitine

In participants without HF, the odds of an association between MCAC and eGFR 15–29 ml/min (CKD stage 4) were not significantly different from those with eGFR≥90 ml/min in the adjusted models (Table 3). In contrast, in participants with HF, eGFR 15–29 ml/min (CKD stage 4) was significantly associated with a higher MCAC than those with normal kidney function. In this group, CKD stage 4 was associated with 1.28-fold (95% CI: 1.15–1.42, *P* < 0.001) higher odds of increasing MCAC as compared to normal kidney function in the fully adjusted model (Table 3).

#### Long-chain acylcarnitine

In participants without HF, CKD stage 4 was associated with significant odds of lower LCAC, whereas in participants with HF, the odds of association of CKD stage 4 with lower LCAC weakened (toward null) in adjusted models. In those without HF, CKD stage 4 was associated with 0.75-fold (95% CI: 0.65–0.86, *P* < 0.001), whereas in those with HF, CKD stage 4 was associated with 0.84-fold (95% CI: 0.80–0.99, *P* = 0.035) lower odds of increasing LCAC than normal kidney function after adjustments (Table 3).

### Alterations of AC by HF subtypes

#### Short-chain acylcarnitine

**Fig. 3:**
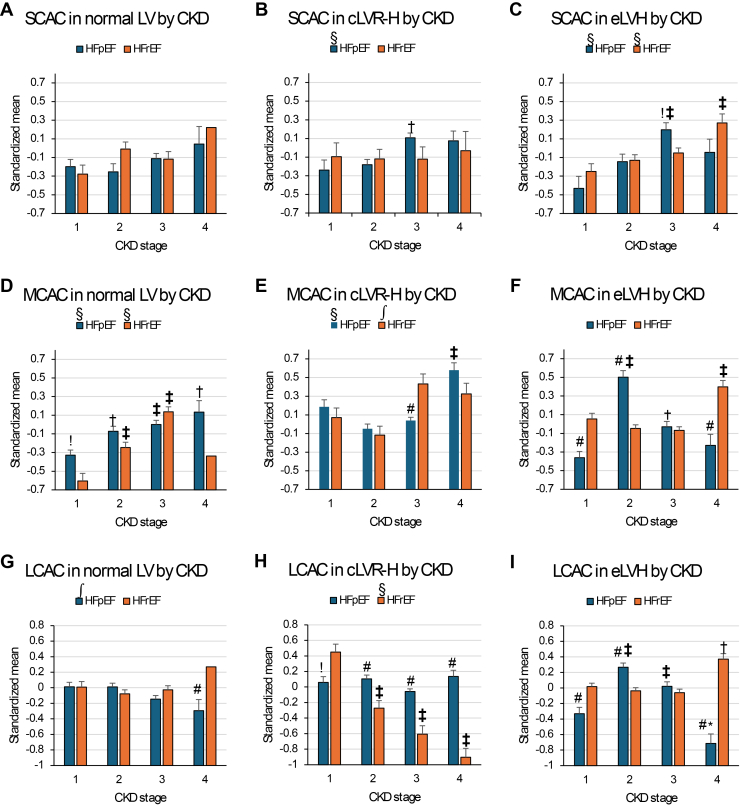
Comparing various chain acylcarnitines in HFpEF versus HFrEF and by left ventricular configuration and CKD stage in patients with heart failure. Panel A refers to mean levels of SCAC in heart failure with normal LV configuration by CKD and heart failure subtype, Panel B to that in cLVR-H by CKD and heart failure subtype, and Panel C to that in eLVH by CKD and heart failure subtype. Panel D refers to mean levels of MCAC in heart failure with normal LV configuration by CKD and heart failure subtype, Panel E to that in cLVR-H by CKD and heart failure subtype, and Panel F to that in eLVH by CKD and heart failure subtype. Panel G refers to mean levels of LCAC in heart failure with normal LV configuration by CKD and heart failure subtype, Panel H to that in cLVR-H by CKD and heart failure subtype, and Panel I to that in eLVH by CKD and heart failure subtype. SCAC, short-chain acylcarnitine; MCAC, medium-chain acylcarnitines; LCAC, long-chain acylcarnitine; CKD, chronic kidney disease; HFpEF, heart failure with preserved ejection fraction; HFrEF, heart failure with reduced ejection fraction; cLVR-H, concentric left ventricular remodeling or hypertrophy; eLVH, eccentric left ventricular remodeling. Values are men and standard error. ∗*P* = 0.013 compared to CKD stage 1; †*P* ≤ 0.01 compared to CKD stage 1; ‡*P* ≤ 0.001 compared to CKD stage 1; !*P* ≤ 0.01 compared with HFrEF within same CKD stage; #*P* ≤ 0.001 compared with HFrEF within same CKD stage; ∫*P* ≤ 0.01 trend by CKD; §*P* ≤ 0.001 trend by CKD.

In HF with normal LV configuration, the mean of SCAC increased from CKD stage 1 to stage 4 in both HFpEF and HFrEF, but the increase did not reach statistical significance. Mean of SCAC in patients with HFpEF and HFrEF was not statistically different at any CKD stage (Fig. 3A). In HF with cLVR or cLVH, mean of SCAC increased from stage 1 to stage 4 of CKD in HFpEF, so that its level at CKD stage 3 was significantly higher than CKD stage 1. SCAC did not significantly differ by CKD stage in HFrEF (Fig. 3B). In HF with eLVH, there was a significant increase in mean SCAC from CKD stage 1 to stage 4 in both HFpEF and HFrEF, so that its level at CKD stage 3 in HFpEF and at CKD stage 4 in HFrEF was significantly higher than CKD stage 1. Mean SCAC at CKD stage 3 was higher in HFpEF than in HFrEF (Fig. 3C).

#### Medium-chain acylcarnitine

In HF with normal LV configuration, the mean of MCAC significantly increased from CKD stage 1 to stage 4 in both HFpEF and HFrEF. In HFpEF, the MCAC was significantly higher in CKD stage 2–4 compared to CKD stage 1, and in HFrEF, it was significantly higher in CKD stage 3 compared to stage 1. Only at CKD stage 1 was MCAC higher in HFpEF, whereas there were no differences in MCAC between HFpEF and HFrEF in CKD stage 2–4 (Fig. 3D). In HF with cLVR or cLVH, there were significant increasing trends in MCAC by worsening CKD stage, but only MCAC in CKD stage 4 reached statistically significant difference compared to CKD stage 1 in HFPEF. At CKD stage 3, MCAC was higher HFrEF than HFrEF with cLVR or cLVH (Fig. 3E). In HF with eLVH, MCAC was higher in CKD stages 2 and 3 compared to stage 1 in HFpEF, whereas in HFrEF, it was only higher in CKD stage 4 compared to stage 1. At CKD stage 2, MCAC was higher in HFpEF than in HFrEF; at CKD stage 3 it was not significantly different, but in CKD stage 4, the level was higher in HFrEF than in HFpEF (Fig. 3F).

#### Long-chain acylcarnitine

In HF with normal LV configuration, the mean of LCAC significantly decreased from CKD stage 1 to stage 4 in HFpEF but not in HFrEF. Mean of LCAC was significantly lower at CKD stage 4 compared to stage 1 in HFpEF. In HF with cLVR or cLVH, LCAC decreased significantly from CKD stage 1 to stage 4 in HFrEF but not in HFPEF. LCAC was lower in CKD stages 2–4 than in CKD stage 1 in HFrEF. The level was higher in HFrEF than in HFpEF at CKD stage 1 but lower at CKD stage 2–4 in HF with cLVR or cLVH (Fig. 3H). In HF with eLVH, LCAC was higher at CKD stage 2 and 3 but lower at stage 4 compared to CKD stage 1 in HFpEF, whereas in HFrEF with eLVH, the level was higher in CKD stage 4 compared to CKD stage 1. LCAC was higher in HFpEF than in HFrEF at CKD stage 2, whereas it was lower in HFpEF than HFrEF at CKD stage 4 in HF with eLVH (Fig. 3I).

## Discussion

We observed significant differences in the patterns of AC that are unique to CKD, HF, and the combination of HF and CKD, implying a spectrum of FAO defects that are unique to each and the combined entity. First, we noted an increase in SCAC by worsening CKD stage. The SCAC increase by worsening CKD stage was significantly higher in patients without HF thanthose with HF. Therefore, the mean of SCAC was significantly lower in HF at CKD stage 4 than those without HF in the same stage. Among patients with HF, those with eLVH and not cLVH had a significant increase in SCAC from CKD stage 1 to CKD stage 4, both in HFpEF and HFrEF. There was also a significant increase in the mean MCAC by worsening CKD stage. The MCAC increase by CKD stage was significantly higher in patients with HF than those with HF. Therefore, the mean level was significantly higher at CKD stage 4 than in those without HF. Among patients with HF, there was a significant increase in MCAC in patients with normal LV configuration and those with cLVR or cLVH from CKD stage 1 to stage 4 both in HFpEF and HFrEF. Whereas in HF with eLVH, only HFrEF had a significant increase in MCAC by CKD progression, so that the level was higher at CKD stage 4 as compared to HFpEF. Finally, the mean LCAC significantly declined in both groups with and without HF, but the slope of the decline was mitigated in the HF group. A steeper decline in those without HF led to a significantly lower mean LCAC in patients without HF than in those without HF. Among patients with HF, those with HFpEF and normal LV configuration had significant decrease in LCAC from CKD stage 1 to stage 4, whereas there were no changes by CKD stage in HFrEF. Among HF with cLVR or cLVH, there was a significant decrease in LCAC from CKD stage 1 to stage 4 in HFrEF but not in HFrEF. Among HF with eLVH, LCAC was higher at CKD stage 4 compared to stage 1 in HFrEF, whereas in HF with eLVH, LCAC declined from CKD stage 2–4 in HFpEF. The significant associations in the fully adjusted models were independent of background variables.

In a case-control study, dilated cardiomyopathy was associated with a 3.1-fold increase in C3 to C18 circulating AC as compared to a healthy control group (10). Ahmed *et al.* showed increased MCAC and LCAC scores with all-cause mortality and hospitalization in patients with HF (12). In a case-control study, Hunter *et al.* showed an association between increased LCAC and worse disease severity in HF (23). Ruiz *et al.* showed 22%–79% higher plasma AC in 68 patients with ischemic HF with reduced ejection fraction. They also showed that the abundance of MCAC was correlated with disease severity (24). In a cross-sectional study of 44 patients with aortic stenosis, there was a direct association between C16, C18:1, C18:2, C18, and C26 AC levels and the LVMI (25). In a metabolic study comparing the abundance of MCAC and LCAC, patients with diabetic cardiomyopathy had a significantly higher abundance of plasma MCAC and LCAC than diabetic patients with no cardiomyopathy (13). In a posthoc analysis of the HF-ACTION clinical trial, LCAC inversely and independently predicted all-cause mortality in patients with diabetes (14). These studies are limited by their sample size and lack of stratified analysis by CKD stage, which does not allow inference of HF-specific AC alterations by CKD stage. To the best of our knowledge, our study is the first to show HF-specific alterations in various-chain AC according to worsening CKD stages.

Under normal physiological conditions, ∼70% of the myocardial energy requirement is met by the β-oxidation of long-chain fatty acids and the rest from glucose (26). Cardiomyocytes convert fatty acids to long-chain fatty acyl-CoA esters by acyl-CoA synthetase long-chain family of enzymes (27). Fatty acyl-CoA esters are then transported to the mitochondria for β-oxidation via mitochondrial AC transporters. Mitochondrial transporters include carnitine palmitoyltransferase-1 and 2 (CPT1 and 2) (27, 28). CPT1 activity is regulated by the cytosolic level of malonyl-CoA, which is regulated by acetyl-CoA carboxylase and malonyl-CoA decarboxylase (29). Acetyl-CoA carboxylase phosphorylation decreases malonyl-CoA levels and improves β-oxidation. Oxidation of each palmitate molecule produces approximately 129 ATPs (30), which is a robust source of cardiac energy metabolism that is more efficient than myocardial glucose oxidation (30, 31, 32). In cardiomyocytes, short-chain fatty acids do not depend on CPT1 for mitochondrial transfer. Therefore, in failing hearts with impaired oxidation of long-chain fatty acids due to reduced CPT1, ATP production shifts from oxidation of long-chain fatty acids to glucose oxidation (33) and utilization of short-chain fatty acids and ketone bodies for energy production (34). Kidneys are also able to oxidize FFAs from 6 to 10 carbons in length without needing the carnitine shuttle, whereas the heart and skeletal muscles depend on the carnitine shuttle for mitochondrial transfer of medium- and long-chain fatty acids (35).

In a rodent study, rats with high running capacity and lifespan compared to their low-capacity counterparts exhibited significantly higher LCAC in plasma and muscle with exercise, while their MCAC in muscle remained relatively lower than their LCAC (36). These findings suggest that inefficiency of β-oxidation could be in part due to decreased mitochondrial transport of MCAC, lower efficiency of energy production by oxidation of MCAC as compared to LCAC, as well as low abundance of extramitochondrial LCAC. In a lipidomic study of CKD, we observed a similar pattern, characterized by increased MCAC and decreased LCAC by the worsening stage of CKD (15), suggesting impaired β-oxidation due to MCAC accumulation and decreased extramitochondrial upstream LCAC substrates.

In CKD, the metabolism of SCAC is disrupted, leading to increased plasma levels (37, 38) in part because of their decreased clearance and impaired mitochondrial β-oxidation, which can further promote CKD progression (39, 40, 41). Unlike LCAC and MCAC, myocardium does not rely on CPT1/CPT2 for mitochondrial influx of short chain fatty acids. In failing hearts, where β-oxidation of medium- and long-chain fatty acids is impaired due to CPT1/CPT2 inactivity, short-chain fatty acids therefore become a preferred substrate. The relatively attenuated increase in SCAC across CKD stages in HF participants—compared with their non-HF counterparts—likely reflects increased myocardial utilization of short-chain fatty acids in failing hearts. The fact that SCAC still increased significantly across CKD stages in both HFpEF and HFrEF supports the additional contribution of decreased renal clearance with CKD progression.

The increase in MCAC due to CKD progression aligns with our previous report of lipidomic alterations in CKD (15). In CKD without HF, plasma MCAC rises in part due to reduced renal clearance of MCAC and in part because of impaired tubular β-oxidation of medium-chain fatty acids due to tubular atrophy and tubulointerstitial fibrosis. In compensated hearts, cardiomyocytes continue to oxidize medium-chain fatty acids for energy production, but in failing hearts, the reduced myocardial capacity for medium-chain β-oxidation leads to further MCAC accumulation beyond the level observed in CKD without HF. With progressive CKD complicated by HF, MCAC accumulates even further as a result of myocardial CPT1/CPT2 inactivity superimposed on the cumulating effect of failing kidneys—accounting for the steeper MCAC slope across CKD stages in HF compared with non-HF participants. We observed increasing MCAC trends across CKD stages in both HFpEF and HFrEF among participants with cLVR or cLVH, consistent with reduced renal clearance. Notably, in HF with eLVH, HFrEF showed significantly higher MCAC than HFpEF at CKD stage 4, likely reflecting the higher LVMI observed in HFrEF at CKD stage 4.

Similarly, we observed a continued decline in LCAC due to CKD progression in patients without HF, consistent with our previous report (15). The decline was attenuated in HF mainly due to increasing LCAC by CKD progression in HFrEF with eLVH and a higher LVMI. Decreased plasma LCAC with CKD progression may be explained by reduced upstream synthesis, accelerated oxidation of long-chain fatty acids to medium-chain fatty acids, or contributions from other factors such as insulin resistance, physical activity, and dietary supplementation. However, evidence suggests that CKD progression promotes downregulation of acyl-CoA synthetase long-chain family enzymes (ACSL) (42), likely due to the detrimental effects of the uremic milieu on enzyme function and reduced carnitine shuttle substrates, both of which impair β-oxidation. This impairment is most pronounced at advanced CKD stages, diminishing the conversion of long-chain fatty acids to LCAC and contributing to reduced LCAC abundance and impaired β-oxidation of long-chain fatty acids, a systemic effect of advanced CKD. In the absence of HF, relatively intact myocardial CPT1/CPT2 activity continues to drive cytosol-to-mitochondrial influx of LCAC, further lowering circulating LCAC. In contrast, CPT1/CPT2 inactivity in failing myocardium prevents this mitochondrial influx, partly explaining the systemic accumulation of LCAC seen in advanced CKD complicated by HF. In a comparison of 282 patients with HFpEF with 279 patients with HFrEF, Hunter *et al.* reported a similarly higher level of LCAC in HFrEF and attributed that to a more severe decompensated HF (23). However, that study was not stratified by CKD stage. Our findings are partially consistent with Hunter’s observation in that LCAC was higher in HFrEF than in HFpEF, but we observed that only in HF with eLVH at CKD stage 4. Lower LCAC in HFpEF with cLVR or cLVH and at earlier stage of CKD might be reflection of a less severe HF stage.

This study has several strengths. To our knowledge, this is the first study to investigate the AC levels at various CKD stages in patients with and without HF. The sample size was relatively large, allowing for a proper subgroup and stratified analysis with reasonable power. Systematic data collection and uniform covariates were allowed for proper multivariable adjustments. State-of-art mass spectrometry quantification of AC with a tight coefficient of variation contributes to accurate measurements. This study has some limitations. The observational nature of this study did not allow us to infer causality or establishing mechanism of disease, rather the associations may reflect suggestive mechanisms. As the matching variable was limited to CKD stage, it was constrained by limited availability of stored plasma samples among potential controls and by inherence baseline imbalances, matching was therefore not one-to-one. However, the multivariate models with adjustment for such covariates suggested that alterations of various chain AC by CKD stage were independent of these covariates or their baseline imbalanced distribution by HF. Each patient had only one measurement of AC, and the effect of the longitudinal time-varying effect of AC on outcomes was not explored. Although our inference was based on subgroups of AC due to their similarities in metabolism and mitochondrial transport mechanism (9), individual AC species may still have unique metabolic steps and biological roles, necessitating study with larger sample size for the subgroup analyses. Residual confounders may not have been adjusted in the fully adjusted models. Although the changes in acylcarnitines are likely the reflection of fatty acid oxidation at varying stages of failing organs, the contribution of other factors such as insulin resistance, physical activity, dietary supplementation, and amino acid degradation (43) cannot be ruled out. The observational nature of our study did not allow measuring myocardial AC in participants. However, there is growing evidence to suggest that with failing heart and worsening LVH, plasma AC concentrations may reflect the AC profile in cardiac tissue (44). This was a single-center observation, and similar studies need to be conducted to extrapolate the findings to the general population of patients with CKD-HF.

This study had several important clinical implications. These findings suggest that in CKD-HF, β-oxidation of medium- and long-chain fatty acids is shifted toward β-oxidation of short-chain fatty acids, which is relatively less efficient (in terms of ATP production) owing to the lower carbon numbers. Furthermore, the accumulation of MCAC with CKD progression disproportionate to those without HF may lead to poorer outcomes due to their arrhythmogenic properties via modulation of ionic currents, especially increased influx of K^+^ currents (45), increased intracellular Ca^2+^ promoting apoptosis and cell death (46), and activation of proinflammatory pathways (47). For a long time, the risk factors for HF were limited to traditional risk factors (6). In this study, we provide evidence for the derailment of energy metabolism in the clinical syndrome of HF with CKD progression. AC are modifiable risk factors; hence, interventions aimed at improving the efficiency of mitochondrial β-oxidation may improve cardiovascular clinical outcomes in the CKD–HF complex.

In this study, we noted an HF-specific pattern of increased SCAC and MCAC but variable LCAC alterations by HF subtype as CKD progressed. Less severe decline in LCAC by CKD stage in HF was driven by high LCAC in HFrEF with eLVH and high LVMI at CKD stage 4, suggesting that higher LCAC may have a worse prognostic value with CKD and HF progression. As AC levels reflect the efficiency of β-oxidation, interventions aimed at improving β-oxidation may prevent metabolic HF, mitigate the clinical course, or improve the clinical outcomes in the CKD–HF complex.

## Data availability

Research data supporting this publication are in possession of corresponding author and can be shared with a reasonable analytical plan.

## Supplemental data

This article contains supplemental data.

## Ethical Compliance

All procedures performed in studies involving human participants were in accordance with the ethical standards of the institutional and/or national research committee and with the 1964 Helsinki Declaration and its later amendments or comparable ethical standards.

## Conflicts of interest

The authors declare that they have no conflicts of interest with the contents of this article.
