# Supplementary material for: Plasma acylcarnitine profiling reveals heart failure-specific fatty acid oxidation signature in chronic kidney disease
Source: J Lipid Res. 2026 May 29;67(7):101072. doi: 10.1016/j.jlr.2026.101072 (PMC13315848; doi:10.1016/j.jlr.2026.101072)
Supplement: Supplemental Data [file mmc1.docx]

**Supplement Table 1:** ICD-9 and ICD-10 codes for selection of heart failure:

I50.814, I50.9, I50.1, I50.20, I50.30, I50.40, I50.813, I50.810, I50.83, I50.84, I50.812, I50, I50.811, I50.82, I50.89, I50.43, I50.41, I50.42, I50.32, I50.31, I50.33, I50.21, I50.22, I50.23, I50.8, I13.0, I11.0, I13.2, I50.81, I09.81, I09.89, 428.0, 428.1, 428.2, 428.3, 428.4, 428.9, 428.43, 428.40, 428.41, 428.42, 428.32, 428.30, 428.31, 428.33, 428.20, 428.21, 428.22, 428.23, 428, 404.11, 404.91, 404.01, 402.11, 402.91, 402.01, 404.93, 404.03, 404.13, 398.91

**Supplemental Table 2:** List of authenticated internal standards

| Component (Isotopic Label, Enrichment) | Purity | Gravimetric Target Concentration (uM) | Concentration by Gravimetry (uM) | Analyzed Concentration (uM) |
| --- | --- | --- | --- | --- |
| L-Carnitine (N-trimethyl-D_9_, 98%) | 99.7% | 152.0 | 157.5 | 152.1 ± 13.3 |
| L-Acetylcarnitine (N-methyl-D_3_, 98%) | 99.5% | 38.0 | 38.1 | 37.4 ± 2.7 |
| L-Propionylcarnitine (N-N-methyl-D3, 98%) | 98.1% | 7.6 | 7.6 | 7.8 ± 0.6 |
| L-Butyrylcarnitine (N-methyl-D_3_, 98%) | 98.7% | 7.6 | 7.6 | 7.4 ± 0.2 |
| L-Isovalerylcarnitine (N-trimethyl-D_9_, 98%) | >98% | 7.6 | 7.6 | 7.5 ± 0.2 |
| L-Octanoylcarnitine (N-methyl-D_3_, 98%) | 99.5% | 7.6 | 7.6 | 7.5 ± 0.2 |
| L-Myristoylcarnitine (N-trimethyl-D_9_, 98%) | >98% | 7.6 | 7.6 | 7.2 ± 0.3 |
| L-Palmitoylcarnitine (N-methyl-D_3_, 98%) | >98% | 15.2 | 15.2 | 14.9 ± 0.5 |

**Supplemental Table 3:** Measured carnitine and acylcarnitines and their coefficient of variation of measurements.

| Class | Sub-class | Compound by carbon number and number of double bonds in acyl chain | Coefficient of variation (%) |
| --- | --- | --- | --- |
| Carnitines | Carnitine | L-Carnitine | 4.6 |
|  | Acylcarnitines (AC) | C(2:0) | 3.5 |
|  |  | C(3:0) | 1.6 |
|  |  | C(4:0) | 2.6 |
|  |  | C(5:0) | 2.1 |
|  |  | C(5-DC) | 8.9 |
|  |  | C(6:0) | 4.6 |
|  |  | C(8:1) | 3.9 |
|  |  | C(8:0) | 2.0 |
|  |  | C(10:1) | 13.1 |
|  |  | C(10:0) | 11.5 |
|  |  | C(12:0-OH) | 10.6 |
|  |  | C(12:1) | 14.1 |
|  |  | C(12:0) | 18.2 |
|  |  | C(14:0-OH) | 11.4 |
|  |  | C(14:1) | 8.5 |
|  |  | C(14:0) | 3.5 |
|  |  | C(14:2) | 19.5 |
|  |  | C(16:0-OH) | 15.3 |
|  |  | C(16:1) | 9.3 |
|  |  | C(16:0) | 2.1 |
|  |  | C(18:2-OH) | 9.6 |
|  |  | C(18:2) | 5.6 |
|  |  | C(18:1) | 5.7 |
|  |  | C(18:0) | 9.5 |
|  |  | C(20:4) | 10.2 |
|  |  | C(20:3) | 16.3 |
|  |  | C(20:2) | 11.4 |
|  |  | C(20:1) | 11.4 |

**Supplement Table 4:** Mean of absolute individual acylcarnitines by stage of chronic kidney disease (CKD) and heart failure

| Analyte | Heart failure | CKD 1  Mean (95% CI) (µmol/L) | CKD 2  Mean (95% CI)  (µmol/L) | CKD 3  Mean (95% CI)  (µmol/L) | CKD 4  Mean (95% CI)  (µmol/L) |
| --- | --- | --- | --- | --- | --- |
| L-Carnitine | No | 47.59 (43.87 – 51.31) | 48.75 (46.65 – 50.84) | 55.52 (52.22 – 58.49) | 63.02 (55.48 – 70.56) |
|  | Yes | 54.42 (50.54 – 58.31) | 69.19 (51.87 – 86.52) | 70.45 (66.26 – 74.63) | 73.23 (60.68 – 85.78) |
| AC2:0 | No | 10.57 (9.40 – 11.74) | 13.37 (12.22 – 14.52) | 15.06 (13.70 – 16.41) | 17.62 (14.74 – 20.51) |
|  | Yes | 13.29 (11.02 – 15.56) | 15.13 (13.82 – 16.44) | 20.89 (19.05 – 22.73) | 28.00 (19.44 – 36.55) |
| AC3:0 | No | 0.526 (0.469 – 0.582) | 0.622 (0.576 – 0.667) | 0.726 (0.669 – 0.783) | 0.895 (0.743 – 1.046) |
|  | Yes | 0.627 (0.554 – 0.700) | 0.864 (0.526 – 1.202) | 0.902 (0.828 – 0.976) | 0.893 (0.738 – 1.050) |
| AC4:0 | No | 0.257 (0.233 – 0.281) | 0.324 (0.302 – 0.346) | 0.429 (0.397 – 0.462) | 0.739 (0.568 – 0.910) |
|  | Yes | 0.330 (0.290 – 0.369) | 0.381 (0.355 – 0.407) | 0.518 (0.481 – 0.554) | 0.625 (0.519 – 0.732) |
| AC5:0 | No | 0.181 (0.157 – 0.205) | 0.195 (0.180 – 0.209) | 0.245 (0.226 – 0.264) | 0.355 (0.306 – 0.404) |
|  | Yes | 0.186 (0.161 – 0.211) | 0.219 (0.200 – 0.238) | 0.261 (0.236 – 0.286) | 0.281 (0.239 – 0.322) |
| AC5-DC | No | 0.009 (0.008 – 0.010) | 0.012 (0.010 – 0.013) | 0.019 (0.017 – 0.022) | 0.024 (0.019 – 0.029) |
|  | Yes | 0.011 (0.009 – 0.013) | 0.015 (0.014 – 0.017) | 0.025 (0.021 – 0.028) | 0.040 (0.029 – 0.051) |
| AC6:0 | No | 0.069 (0.059 – 0.080) | 0.080 (0.073 – 0.087) | 0.100 (0.090 – 0.112) | 0.127 (0.087 – 0.125) |
|  | Yes | 0.094 (0.076 – 0.112) | 0.129 (0.098 – 0.161) | 0.129 (0.116 – 0.143) | 0.218 (0.088 – 0.347) |
| AC8:0 | No | 0.171 (0.151 – 0.192) | 0.205 (0.187 – 0.223) | 0.236 (0.213 – 0.259) | 0.334 (0.149 – 0.521) |
|  | Yes | 0.224 (0.167 – 0.281) | 0.331 (0.210 – 0.452) | 0.286 (0.250 – 0.322) | 0.703 (0.030 – 1.438) |
| AC8:1 | No | 0.316 (0.285 – 0.348) | 0.357 (0.333 – 0.380) | 0.463 (0.424 – 0.503) | 0.610 (0.519 – 0.701) |
|  | Yes | 0.392 (0.349 – 0.435) | 0.474 (0.436 – 0.511) | 0.612 (0.560 – 0.664) | 0.800 (0.655 – 0.944) |
| AC10:0 | No | 0.263 (0.232 – 0.294) | 0.307 (0.281 – 0.334) | 0.338 (0.300 – 0.375) | 0.473 (0.231 – 0.714) |
|  | Yes | 0.343 (0.262 – 0.424) | 0.489 (0.337 – 0.641) | 0.440 (0.384 – 0.496) | 0.991 (0.063 – 1.919) |
| AC10:1 | No | 0.150 (0.129 – 0.170) | 0.168 (0.155 – 0.181) | 0.219 (0.196 – 0.242) | 0.251 (0.186 – 0.317) |
|  | Yes | 0.191 (0.159 – 0.222) | 0.246 (0.210 – 0.281) | 0.271 (0.241 – 0.301) | 0.475 (0.210 – 0.741) |
| AC12:0-OH | No | 0.006 (0.005 – 0.007) | 0.007 (0.006 – 0.008) | 0.010 (0.009 – 0.011) | 0.011 (0.009 – 0.014) |
|  | Yes | 0.007 (0.006 – 0.008) | 0.009 (0.008 – 0.010) | 0.013 (0.012 – 0.015) | 0.019 (0.014 – 0.024) |
| AC12:0 | No | 0.041 (0.036 – 0.046) | 0.050 (0.045 – 0.054) | 0.062 (0.055 – 0.070) | 0.070 (0.049 – 0.091) |
|  | Yes | 0.057 (0.045 – 0.068) | 0.093 (0.059 – 0.127) | 0.092 (0.076 – 0.109) | 0.228 (0.040 – 0.497) |
| AC12:1 | No | 0.038 (0.029 – 0.047) | 0.043 (0.038 – 0.048) | 0.048 (0.041 – 0.054) | 0.048 (0.037 – 0.060) |
|  | Yes | 0.052 (0.043 – 0.061) | 0.068 (0.056 – 0.081) | 0.086 (0.073 – 0.099) | 0.150 (0.054 – 0.246) |
| AC14:0-OH | No | 0.008 (0.007 – 0.009) | 0.010 (0.009 – 0.011) | 0.014 (0.012 – 0.015) | 0.011 (0.008 – 0.014) |
|  | Yes | 0.009 (0.007 – 0.011) | 0.012 (0.011 – 0.014) | 0.015 (0.013 – 0.017) | 0.013 (0.011 – 0.016) |
| AC14:0 | No | 0.039 (0.035 – 0.044) | 0.048 (0.045 – 0.052) | 0.056 (0.051 – 0.061) | 0.055 (0.042 – 0.067) |
|  | Yes | 0.054 (0.047 – 0.061) | 0.084 (0.066 – 0.101) | 0.078 (0.070 – 0.086) | 0.098 (0.046 – 0.151) |
| AC14:1 | No | 0.067 (0.058 – 0.076) | 0.085 (0.076 – 0.094) | 0.092 (0.081 – 0.103) | 0.106 (0.079 – 0.133) |
|  | Yes | 0.096 (0.080 – 0.112) | 0.161 (0.118 – 0.203) | 0.155 (0.134 – 0.176) | 0.229 (0.091 – 0.366) |
| AC14:2 | No | 0.036 (0.030 – 0.041) | 0.043 (0.038 – 0.048) | 0.051 (0.043 – 0.058) | 0.064 (0.045 – 0.084) |
|  | Yes | 0.052 (0.042 – 0.062) | 0.081 (0.066 – 0.097) | 0.083 (0.073 – 0.093) | 0.123 (0.053 – 0.193) |
| AC16:0-OH | No | 0.027 (0.019 – 0.036) | 0.024 (0.021 – 0.026) | 0.035 (0.027 – 0.042) | 0.028 (0.016 – 0.039) |
|  | Yes | 0.025 (0.020 – 0.032) | 0.025 (0.021 – 0.029) | 0.024 (0.020 – 0.028) | 0.025 (0.014 – 0.035) |
| AC16:0 | No | 0.555 (0.466 – 0.644) | 0.589 (0.538 – 0.639) | 0.633 (0.573 – 0.692) | 0.585 (0.447 – 0.723) |
|  | Yes | 0.592 (0.529 – 0.654) | 0.675 (0.609 – 0.740) | 0.730 (0.663 – 0.796) | 0.761 (0.630 – 0.893) |
| AC16:1 | No | 0.165 (0.130 – 0.200) | 0.206 (0.176 – 0.236) | 0.214 (0.183 – 0.245) | 0.208 (0.128 – 0.289) |
|  | Yes | 0.203 (0.159 – 0.247) | 0.288 (0.226 – 0.350) | 0.298 (0.244 – 0.351) | 0.379 (0.204 – 0.554) |
| AC18:0 | No | 0.239 (0.198 – 0.279) | 0.254 (0.230 – 0.278) | 0.263 (0.235 – 0.291) | 0.254 (0.182 – 0.325) |
|  | Yes | 0.230 (0.203 – 0.256) | 0.274 (0.244 – 0.305) | 0.279 (0.252 – 0.307) | 0.280 (0.223 – 0.336) |
| AC18:1 | No | 0.873 (0.737 – 1.010) | 0.959 (0.867 – 1.051) | 0.890 (0.804 – 0.976) | 0.910 (0.670 – 1.150) |
|  | Yes | 0.992 (0.873 – 1.112) | 1.152 (1.028 – 1.275) | 1.254 (1.118 – 1.391) | 1.358 (1.094 – 1.623) |
| AC18:2 | No | 0.399 (0.326 – 0.472) | 0.408 (0.370 – 0.447) | 0.414 (0.374 – 0.452) | 0.435 (0.313 – 0.556) |
|  | Yes | 0.476 (0.420 – 0.532) | 0.580 (0.517 – 0.642) | 0.618 (0.554 – 0.681) | 0.640 (0.504 – 0.775) |
| AC18:2-OH | No | 0.018 (0.014 – 0.022) | 0.024 (0.020 – 0.027) | 0.028 (0.024 – 0.032) | 0.023 (0.013 – 0.033) |
|  | Yes | 0.019 (0.016 – 0 .022) | 0.023 (0.020 – 0.027) | 0.026 (0.023 – 0.030) | 0.029 (0.017 – 0.041) |
| AC20:0 | No | 0.055 (0.039 – 0.071) | 0.065 (0.052 – 0.078) | 0.080 (0.063 – 0.097) | 0.100 (0.054 – 0.146) |
|  | Yes | 0.049 (0.040 – 0.059) | 0.078 (0.060 – 0.095) | 0.105 (0.081 – 0.129) | 0.139 (0.085 – 0.194) |
| AC20:1 | No | 0.037 (0.031 – 0.043) | 0.041 (0.037 – 0.045) | 0.038 (0.034 – 0.043) | 0.045 (0.030 – 0.059) |
|  | Yes | 0.034 (0.030 – 0.038) | 0.046 (0.040 – 0.051) | 0.048 (0.041 – 0.054) | 0.051 (0.040 – 0.062) |
| AC20:2 | No | 0.020 (0.017 – 0.024) | 0.021 (0.019 – 0.023) | 0.020 (0.019 – 0.022) | 0.023 (0.017 – 0.028) |
|  | Yes | 0.022 (0.019 – 0.024) | 0.026 (0.024 – 0.029) | 0.029 (0.026 – 0.032) | 0.031 (0.024 – 0.039) |
| AC20:3 | No | 0.011 (0.009 – 0.014) | 0.011 (0.010 – 0.012) | 0.011 (0.010 – 0.012) | 0.011 (0.008 – 0.013) |
|  | Yes | 0.013 (0.012 – 0.015) | 0.016 (0.014 – 0.018) | 0.018 (0.016 – 0.019) | 0.018 (0.013 – 0.022) |
| AC20:4 | No | 0.017 (0.014 – 0.021) | 0.018 (0.016 – 0.020) | 0.019 (0.017 – 0.021) | 0.020 (0.014 – 0.026) |
|  | Yes | 0.023 (0.019 – 0.026) | 0.028 (0.024 – 0.031) | 0.030 (0.027 – 0.034) | 0.032 (0.021 – 0.041) |

**Supplement Table 5**: Comparing mean levels and 95% confidence interval (CI) of L-Carnitine and individual acylcarnitines by stage of chronic kidney disease (CKD) and heart failure after class level sum normalization, logit transformation and z-score standardization

| Analyte (µmol/L) | Heart failure | CKD 1  Mean (95% CI) | CKD 2  Mean (95% CI) | CKD 3  Mean (95% CI) | CKD 4  Mean (95% CI) |
| --- | --- | --- | --- | --- | --- |
| L-Carnitine | No § | 0.28 (0.09 to 0.48) | 0.05 (-0.09 to 0.19) | 0.04 (-0.10 to 0.19)!! | -0.04 (-0.32 to 0.25) |
|  | Yes | 0.23 (0.04 to 0.45) | 0.15 (-0.00 to 0.31) | -0.09 (-0.22 to 0.04) | -0.52 (-0.79 to -0.26) |
| AC2:0 | No § | -0.28 (-0.50 to -0.05) | -0.02 (-0.16 to 0.12) | -0.02 (-0.18 to 0.14)!! | 0.09 (-0.18 to 0.34) |
|  | Yes § | -0.21 (-0.40 to -0.03) | -0.17 (-0.32 to -0.02) | 0.15 (0.02 to 0.28) | 0.42 (0.15 to 0.68) |
| AC3:0 | No § | -0.17 (-0.38 to 0.03) | 0.04 (-0.10 to 0.17) | 0.15 (0.01 to 0.28)!! | 0.30 (-0.08 to 0.69) |
|  | Yes | -0.20 (-0.39 to -0.01) | -0.11 (-0.29 to 0.06) | 0.02 (-0.11 to 0.15) | -0.18 (-0.45 to 0.09) |
| AC4:0 | No § | -0.56 (-0.76 to -0.36)! | -0.21 (-0.33 to -0.09)!! | 0.12 (-0.02 to 0.27)!! | 0.90 (0.50 to 1.29) |
|  | Yes § | -0.44 (-0.63 to -0.24) | -0.30 (-0.43 to -0.16) | 0.01 (-0.10 to 0.11) | 0.29 (-0.03 to 0.56) |
| AC5:0 | No § | -0.10 (-0.33 to 0.12) | -0.04 (-0.17 to 0.09) | 0.19 (0.05 to 0.32) | 0.65 (0.33 to 0.97) |
|  | Yes § | -0.36 (-0.56 to -0.16) | -0.23 (-0.37 to -0.08) | -0.19 (-0.32 to -0.06) | -0.07 (-0.36 to 0.21) |
| AC5-DC | No § | -0.48 (-0.67 to -0.29) | -0.30 (-0.41 to -0.19)! | 0.16 (0.01 to 0.30) | 0.43 (0.18 to 0.69)! |
|  | Yes § | -0.51 (-0.69 to -0.34) | -0.24 (-0.37 to -0.10) | 0.09 (-0.04 to 0.21) | 0.69 (0.43 to 0.94) |
| AC6:0 | No § | -0.26 (-0.46 to -0.05) | -0.13 (-0.25 to -0.02) | 0.01 (-0.14 to 0.16) | 0.12 (-0.22 to 0.47) |
|  | Yes # | -0.16 (-0.37 to 0.05) | -0.05 (-0.21 to 0.11) | -0.00 (-0.13 to 0.12) | 0.35 (0.07 to 0.64) |
| AC8:0 | No § | -0.11 (-0.30 to 0.08) | 0.01 (-0.12 to 0.13) | 0.04 (-0.11 to 0.18) | 0.05 (-0.33 to 0.42) |
|  | Yes | -0.20 (-0.42 to 0.03) | -0.08 (-0.24 to 0.09) | -0.11 (-0.23 to 0.02) | 0.33 (-0.00 to 0.66) |
| AC8:1 | No § | -0.36 (-0.56 to -0.17)! | -0.27 (-0.39 to -0.15)!! | -0.04 (-0.18 to 0.10)!! | 0.31 (0.04 to 0.57) |
|  | Yes § | -0.29 (-0.49 to -0.08) | -0.13 (-0.27 to 0.00) | 0.00 (-0.12 to 0.12) | 0.46 (0.19 to 0.73) |
| AC10:0 | No § | -0.08 (-0.26 to 0.11) | -0.01 (-0.13 to 0.12) | -0.07 (-0.21 to 0.08)! | -0.10 (-0.51 to 0.31) |
|  | Yes | -0.15 (-0.38 to 0.09) | -0.02 (-0.18 to 0.14) | -0.08 (-0.21 to 0.05) | 0.37 (0.04 to 0.70) |
| AC10:1 | No § | -0.24 (-0.44 to -0.04) | -0.14 (-0.26 to -0.02)!! | 0.01 (-0.14 to 0.17)! | -0.05 (-0.42 to 0.32) |
|  | Yes § | -0.19 (-0.40 to 0.02) | -0.07 (-0.21 to 0.08) | -0.05 (-0.18 to 0.08) | 0.47 (0.15 to 0.79) |
| AC12:0-OH | No § | -0.28 (-0.47 to -0.08) | -0.22 (-0.35 to -0.10) | 0.09 (-0.05 to 0.23)!! | -0.02 (-0.40 to 0.36)! |
|  | Yes § | -0.39 (-0.58 to -0.19) | -0.16 (-0.30 to -0.01) | 0.08 (-0.06 to 0.22) | 0.54 (0.27 to 0.80) |
| AC12:0 | No § | -0.21 (-0.39 to -0.03) | -0.15 (-0.28 to -0.02) | -0.06 (-0.22 to 0.11)!! | -0.09 (-0.44 to 0.26) |
|  | Yes | -0.14 (-0.33 to 0.06) | 0.04 (-0.12 to 0.20) | 0.12 (-0.01 to 0.24) | 0.35 (0.04 to 0.67) |
| AC12:1 | No | -0.23 (-0.43 to -0.04) | -0.17 (-0.30 to -0.05)!! | -0.17 (-0.31 to -0.04) | -0.25 (-0.55 to 0.05) |
|  | Yes § | -0.03 (-0.22 to 0.16) | 0.00 (-0.15 to 0.15) | 0.11 (-0.03 to 0.25) | 0.55 (0.25 to 0.84) |
| AC14:0-OH | No § | -0.17 (-0.37 to 0.02) | -0.03 (-0.17 to 0.11) | 0.26 (0.09 to 0.44) | -0.29 (-0.65 to 0.06) |
|  | Yes § | -0.26 (-0.45 to -0.06) | -0.11 (-0.26 to 0.05) | 0.03 (-0.10 to 0.16) | -0.11 (-0.34 to 0.11) |
| AC14:0 | No § | -0.25 (-0.44 to -0.05)!! | -0.08 (-0.19 to 0.04)!! | -0.03 (-0.18 to 0.11)!! | -0.36 (-0.71 to -0.02) |
|  | Yes | -0.07 (-0.27 to 0.14) | 0.14 (-0.03 to 0.30) | 0.00 (-0.14 to 0.14) | 0.06 (-0.25 to 0.38) |
| AC14:1 | No § | -0.22 (-0.40 to -0.04)! | -0.10 (-0.22 to 0.02) | -0.13 (-0.27 to 0.01)!! | -0.21 (-0.54 to 0.13) |
|  | Yes # | -0.08 (-0.28 to 0.12) | 0.08 (-0.08 to 0.24) | 0.08 (-0.06 to 0.22) | 0.33 (0.03 to 0.63) |
| AC14:2 | No § | -0.24 (-0.43 to -0.05)! | -0.18 (-0.30 to -0.05) | -0.16 (-0.31 to -0.01)!! | -0.08 (-0.42 to 0.25) |
|  | Yes § | -0.08 (-0.29 to 0.12) | 0.07 (-0.08 to 0.23) | 0.12 (-0.01 to 0.26) | 0.33 (0.02 to 0.63) |
| AC16:0-OH | No | 0.23 (0.01 to 0.45) | 0.15 (0.03 to 0.26) | 0.19 (0.02 to 0.37) | -0.10 (-0.41 to 0.21) |
|  | Yes | 0.04 (-0.15 to 0.24) | -0.15 (-0.29 to -0.01) | -0.36 (-0.50 to -0.23) | -0.48 (-0.82 to -0.15) |
| AC16:0 | No | 0.06 (-0.18 to 0.29) | 0.09 (-0.04 to 0.22) | 0.03 (-0.12 to 0.18) | -0.40 (-0.74 to -0.06) |
|  | Yes # | 0.03 (-0.17 to 0.23) | -0.06 (-0.20 to 0.09) | -0.15 (-0.028 to -0.02) | -0.14 (-0.51 to 0.23) |
| AC16:1 | No | -0.11 (-0.31 to 0.10) | 0.03 (-0.10 to 0.16) | -0.01 (-0.16 to 0.15)! | -0.30 (-0.66 to 0.07) |
|  | Yes | -0.04 (-0.23 to 0.15) | 0.03 (-0.12 to 0.18) | -0.06 (-0.20 to 0.08) | 0.14 (-0.19 to 0.46) |
| AC18:0 | No | 0.10 (-0.15 to 0.35) | 0.20 (0.07 to 0.33) | 0.06 (-0.10 to 0.23) | -0.26 (-0.59 to 0.06) |
|  | Yes | 0.00 (-0.18 to 0.18) | -0.06 (-0.20 to 0.08) | -0.20 (-0.33 to -0.07) | -0.30 (-0.67 to 0.07) |
| AC18:1 | No | 0.03 (-0.20 to 0.27) | 0.10 (-0.04 to 0.23) | -0.13 (-0.28 to 0.01)!! | -0.47 (-0.83 to -0.11) |
|  | Yes # | 0.09 (-0.11 to 0.29) | 0.02 (-0.13 to 0.17) | -0.05 (-0.19 to 0.08) | 0.05 (-0.30 to 0.40) |
| AC18:2 | No | -0.08 (-0.32 to 0.16) | -0.05 (-0.18 to 0.08)!! | -0.13 (-0.26 to 0.01)!! | -0.43 (-0.79 to -0.07) |
|  | Yes # | 0.12 (-0.07 to 0.31) | 0.11 (-0.03 to 0.25) | 0.01 (-0.12 to 0.15) | 0.01 (-0.35 to 0.38) |
| AC18:2-OH | No | -0.01 (-0.22 to 0.19) | 0.16 (0.03 to 0.29) | 0.24 (0.07 to 0.40) | -0.37 (-0.76 to 0.02) |
|  | Yes # | -0.11 (-0.29 to 0.08) | -0.11 (-0.26 to 0.03) | -0.16 (-0.30 to -0.02) | -0.10 (-0.39 to 0.20) |
| AC20:0 | No # | -0.11 (-0.34 to 0.11) | -0.06 (-0.19 to 0.08) | 0.06 (-0.10 to 0.23) | 0.18 (-0.15 to 0.51) |
|  | Yes § | -0.25 (-0.45 to -0.06) | -0.12 (-0.26 to 0.03) | -0.05 (-0.19 to 0.10) | 0.27 (-0.06 to 0.59) |
| AC20:1 | No | 0.12 (-0.10 to 0.34) | 0.21 (0.08 to 0.34) | -0.02 (-0.17 to 0.13) | -0.15 (-0.50 to 0.20) |
|  | Yes # | -0.11 (-0.31 to 0.08) | -0.02 (-0.17 to 0.12) | -0.14 (-0.28 to -0.00) | -0.10 (-0.45 to 0.25) |
| AC20:2 | No | 0.02 (-0.23 to 0.27) | 0.08 (-0.05 to 0.21)! | -0.10 (-0.25 to 0.04)!! | -0.31 (-0.66 to 0.04) |
|  | Yes § | 0.07 (-0.11 to 0.26) | 0.05 (-0.08 to 0.19) | -0.01 (-0.15 to 0.13) | -0.01 (-0.37 to 0.36) |
| AC20:3 | No | -0.03 (-0.28 to 0.22) | -0.03 (-0.16 to 0.09)!! | -0.20 (-0.35 to 0.05)!! | -0.46 (-0.77 to -0.15)! |
|  | Yes | 0.18 (-0.02 to 0.38) | 0.16 (0.02 to 0.30) | 0.10 (-0.04 to 0.24) | 0.03 (-0.33 to 0.38) |
| AC20:4 | No | -0.12 (-0.35 to 0.12) | -0.11 (-0.24 to 0.01)!! | -0.16 (-0.31 to -0.01)!! | -0.35 (-0.66 to -0.04) |
|  | Yes # | 0.15 (-0.04 to 0.35) | 0.18 (0.03 to 0.32) | 0.07 (-0.08 to 0.21) | -0.02 (-0.39 to 0.35) |

**Note:**

For trend: # p ≤ 0.01, § p ≤0.001,

For comparing mean of acylcarnitine in no heart failure versus heart failure within the same CKD stage: ! p≤0.01, !! p≤0.001
